# Association between discharge teaching quality and caregiving competence among parents of preterm infants at NICU discharge: the mediating role of internal locus of control

**DOI:** 10.3389/fped.2026.1826901

**Published:** 2026-07-23

**Authors:** Jingjing Gong, Fengyun Tian, Qingyan Chi, Aixiang Li, Jing Li, Qianqian Hu

**Affiliations:** Linyi People’s Hospital, Linyi, China

**Keywords:** caregiving competence, discharge teaching quality, internal locus of control, parents, premature infants

## Abstract

**Objective:**

To examine the association between discharge teaching quality and caregiving competence among parents of preterm infants at neonatal intensive care unit (NICU) discharge, and to test whether internal locus of control mediates this association.

**Methods:**

In this cross-sectional study, 409 parents of preterm infants discharged from the NICU of a tertiary hospital in Shandong Province, China, were recruited by convenience sampling between May and October 2025. On the day of discharge, participants completed a demographic questionnaire, the Quality of Discharge Teaching Scale-Parent Version (QDTS-PV), the Internal Locus of Control Scale (ILCS; Levenson IPC subscale I), and the Infant Parenting Inventory (IPI). Pearson correlation analysis was used to examine bivariate associations. Hierarchical multiple regression and PROCESS Model 4 with 5,000 bootstrap samples were used to test the mediating role of internal locus of control.

**Results:**

The average score of caregiving competence was (121.18 ± 24.70). Pearson correlation analysis showed that discharge teaching quality and internal locus of control were significantly positively correlated with caregiving competence; discharge teaching quality was significantly positively correlated with internal locus of control (all *P* < 0.01). In multivariate linear regression analysis, parental individual factors explained 14.8% of the variance in caregiving competence; adding discharge teaching quality increased the explained variance to 29.2%, and further adding internal locus of control increased it to 31.8%. Mediation analysis showed that discharge teaching quality had both a direct positive association with caregiving competence and an indirect association through internal locus of control. The indirect effect was 0.054 (all *P* < 0.001), accounting for 15.0% of the total effect.

**Conclusion:**

Internal locus of control partially mediates the relationship between discharge teaching quality and caregiving competence. These findings suggest that strengthening parents’ internal control beliefs may be an important psychological pathway through which high-quality discharge teaching supports caregiving competence, providing a useful reference for developing targeted discharge education strategies.

## 1 Introduction

According to WHO data (1), approximately 13 million preterm infants are born globally each year, accounting for 10% of all newborns. Due to the immaturity of their organs, preterm infants face long-term complications such as growth retardation and delayed cognitive development even after discharge (2, 3). Parents must not only meet their basic needs but also acquire essential medical care knowledge and skills (4). High-quality discharge teaching may help parents acquire the knowledge and skills required for post-discharge care. However, many parents currently exhibit deficiencies in preterm infant care (5, 6), which not only compromises care quality but also increases the risk of readmission (7). Therefore, enhancing the caregiving competence of parents of preterm infants is crucial. Previous studies (8) have found that educational quality can effectively improve parental caregiving competence, but the specific mechanisms underlying this relationship remain unclear. Identifying the mediating factors involved is of significant importance. Discharge teaching quality is particularly important at the time of NICU discharge, when parents begin to assume primary responsibility for caring for a medically vulnerable infant at home. High-quality discharge teaching may help parents understand their infant's condition, master essential caregiving skills, and prepare for common post-discharge challenges. In this sense, discharge teaching is not only an educational process but also a key clinical intervention that may facilitate parents' adaptation to a new caregiving role. Tajalli et al. (9) demonstrated that psychological factors significantly influence the caregiving competence of preterm infant caregivers. Previous research has shown that internal locus of control is positively associated with self-care capacity (10–12). The concept of “locus of control” originated from Rotter's (13) 1966 social learning theory, representing an individual's belief regarding the attribution of health outcomes (14). In the context of preterm infant care, internal locus of control reflects the extent to which parents believe that their own caregiving behaviors can positively influence their infant's health, recovery, and development. Parents with a stronger internal locus of control may be more likely to engage in active learning, problem-solving, and sustained caregiving efforts, which in turn may promote better caregiving competence. As a malleable belief, internal locus of control can be guided and reinforced through targeted health education (15). Therefore, it is plausible that high-quality discharge teaching may enhance caregiving competence not only directly by providing knowledge and skills, but also indirectly by strengthening parents' internal locus of control.

Transition Theory provides a useful framework for understanding this process. According to Transition Theory, individuals undergoing health-related transitions often experience changes in roles, responsibilities, behaviors, and emotional states, and their transition outcomes are influenced by personal, community, and societal conditions (16). For parents of preterm infants, discharge from the NICU represents a critical transition from hospital-supported care to home-based caregiving. During this transition, parents must adapt to a new role, manage uncertainty, and develop confidence in their ability to care for their infant independently. From this perspective, discharge teaching quality may be considered a transitional support, internal locus of control a key psychological condition, and caregiving competence an important transition outcome.

Although previous studies have examined the relationship between discharge education and parental caregiving outcomes, research on the mediating role of internal locus of control in this association among parents of preterm infants remains limited. In addition, the relevance of Transition Theory to this relationship has not been sufficiently clarified in existing research. Therefore, guided by Transition Theory, the present study aimed to examine the association between discharge teaching quality and caregiving competence among parents of preterm infants at NICU discharge and to test whether internal locus of control mediates this association. By clarifying both the direct and indirect pathways, this study seeks to provide evidence for developing discharge interventions that address not only parents’ practical caregiving needs but also their psychological readiness for home care.

### 1.1 Research hypotheses

*H1*: Discharge teaching quality is positively associated with parental caregiving competence.

*H2*: Discharge teaching quality is positively associated with parents' internal locus of control.

*H3*: Parents' internal locus of control is positively associated with parental caregiving competence.

*H4*: Internal locus of control mediates the association between discharge teaching quality and parental caregiving competence.

## 2 Methods

### 2.1 Participants

This was a single-center cross-sectional study using convenience sampling. Mothers or fathers of preterm infants admitted to the NICU of a tertiary hospital in Shandong Province between May 2025 and October 2025 were selected as study subjects. Inclusion Criteria: (1) parents of preterm infants with gestational age <37 weeks who received NICU treatment post-delivery; (2) parents of preterm infants with NICU hospitalization duration >3 days (17); (3) parents primarily responsible for the infant's care at home; (4) parents with clear consciousness and cognition, possessing basic literacy and communication skills; (5) informed consent and voluntary participation in this study. Exclusion criteria: (1) parents of preterm infants with concomitant genetic metabolic disorders or severe congenital anomalies; (2) parents of preterm infants discharged against medical advice/transferred to another facility or readmitted before meeting discharge criteria; (3) parents who voluntarily withdrew from the study; (4) families in which the primary home caregiver was not a parent or in which the caregiving arrangement did not allow assessment of the target parent population. The sample size was estimated based on the requirements for multivariable regression analysis. According to the formula for general linear regression, with *α* = 0.05, test power (1 − *β*) = 0.8, and moderate effect size, *N* ≥ 50 + 8 m (where m is the number of predictors). This study included 22 independent variables (15 variables in general information and 7 variables across scale dimensions), yielding *N* ≥ 226 (18). Accounting for a 20% sample loss rate, a minimum sample size of 283 cases was required. The study ultimately obtained 409 valid responses. This research was approved by the Ethics Committee of Linyi People's Hospital (Ethics Approval No.: 2025-R-139). All participants signed informed consent forms.

### 2.2 Assessment tools

According to the study framework, discharge teaching quality was considered the independent variable, internal locus of control the mediator, and caregiving competence the dependent variable. The assessment tools used in this study included a general information questionnaire, the Quality of Discharge Teaching Scale–Parent Version (QDTS-PV), the Internality subscale of the Internal, Powerful Others, and Chance (IPC) Scale, and the Infant Parenting Competence Scale (IPS).

#### 2.2.1 General information questionnaire

This questionnaire was developed after review by two NICU nursing specialists and one NICU physician to collect demographic data on preterm infants and their parents. General data for preterm infants included gender, gestational age at birth, birth weight, Apgar scores at 1 min (points) and 5 min (points), gestational age at discharge, discharge weight, and length of hospitalization. General data for parents included age, marital status, education level, occupation, family residence, household economic status, and prior childcare experience.

#### 2.2.2 Quality of discharge teaching scale—parent version (QDTS-PV)

The Quality of Discharge Teaching Scale—Parent Version (QDTS-PV) was developed by Weiss et al. and revised by Chen et al. (19). It comprises three subscales (Content Needed, Content Received, and Delivery) with a total of 18 items. In this study, the total QDTS-PV score was calculated according to the original scoring approach: items in the Content Needed subscale were not included in the total score. Thus, the total score was obtained by summing the Content Received and Delivery subscale scores. In this study, the QDTS-PV total score was used to represent parents' discharge teaching quality. All items are rated on a 0–10 scale. The possible total-score range was 0–180; higher scores indicate better discharge teaching quality. In the present study, Cronbach's alpha for the QDTS-PV was 0.964.

#### 2.2.3 Internal locus of control scale (ILCS)

Levenson (20) developed the Internal, Power, and Chance Orientation Scale (IPC Scale), which comprises three subscales. Because the present study focused on parents' beliefs about their own ability to influence caregiving outcomes through their actions, only the Internality (I) subscale was used. Here, internal locus of control was conceptualized as a psychological resource reflecting the extent to which parents believed that their own efforts and behaviors could affect the health and care of their preterm infant. Higher scores indicate a stronger tendency toward internal locus of control characteristics. The scale comprises 8 items, each rated on a 7-point scale ranging from Strongly Disagree (−3) to Strongly Agree (+3). During calculation, 24 points are added to offset negative scores. Thus, the scale scores range from 0 to 48 points. Higher scores indicate a stronger tendency toward internal locus of control characteristics. In this study, the Cronbach's alpha coefficient for this scale was 0.887.

#### 2.2.4 Infant parenting inventory (IPI)

Developed by Wei et al. (21) based on the Knowledge-Belief-Action Theory, the Infant Parenting Inventory is a comprehensive and well-established questionnaire for assessing newborn care competencies. In this study, the IPI total score was used to represent parental caregiving competence. The questionnaire comprises 43 items across three dimensions: caregiving knowledge (26 items), caregiving behavior (12 items), and caregiving attitude (5 items). It employs a 5-point Likert scale ranging from 0 (completely unaware/unable to perform) to 4 (fully aware/fully capable), yielding a total score between 0 and 172. Higher scores indicate stronger parenting competencies. In this study, the Cronbach's alpha coefficient for this scale was 0.972.

### 2.3 Data collection

Based on research objectives and clinical experience, all investigators underwent standardized training prior to the survey to ensure data collection accuracy. Before data collection, the purpose and significance of this study were explained to parents of preterm infants to secure their understanding and cooperation. Paper-based questionnaires were used for data collection, distributed 3–4 h before the infants' discharge. The parent primarily responsible for post-discharge care completed the anonymous questionnaire, which was collected 30 min later. After collection, the questionnaires were checked for completeness and response quality. Questionnaires with substantial missing data, obvious response sets, or patterned responses were considered invalid and excluded from the analysis. A total of 432 questionnaires were distributed, with 409 ultimately determined to be valid, yielding an effective response rate of 94.68%. Data were double-entered using Epi Data 3.1, verified for consistency, and then imported into SPSS for analysis.

### 2.4 Statistical analysis

Data were analyzed using SPSS version 23.0. Normally distributed continuous variables are presented as mean ± standard deviation (*x¯* ± *s*) and categorical variables as frequencies and percentages (%). Common method bias was assessed using the Harman single-factor method. Correlations between variables were evaluated via Pearson correlation analysis. The mediating effect of internal locus of control on the relationship between discharge teaching quality and caregiving competence was tested using the bootstrap method with 5,000 resamples. All tests employed two-tailed significance levels. *P* < 0.05 was considered statistically significant.

## 3 Results

### 3.1 Common method bias test

A Harman single-factor test was conducted to assess common method bias arising from self-report questionnaires. Results indicate that the first principal component explains 21.14% of variance, significantly below the 40% critical threshold. This suggests that common method bias was not a serious concern in this study.

### 3.2 Univariate analysis of parenting competence among parents of preterm infants

Univariate analysis showed that caregiving competence differed significantly according to parental role, education level, occupation, household economic status, and prior childcare experience (all *P* < 0.05). No significant differences were observed for age, marital status, residence, infant sex, gestational age at birth, birth weight, Apgar scores, gestational age at discharge, discharge weight, or length of hospital stay (all *P* > 0.05) (Tables 1, 2).

**Table 1:** Univariate analysis of parenting competence in parents of preterm infants based on general demographic characteristics.

Variable	Group	*N* (%)	*M* ± *SD*	*t*/*F*	*P*
Parental Role	Father	203 (49.6)	117.20 ± 26.49	3.268	0.001
	Mother	206 (50.4)	125.10 ± 22.17		
Age (years)				2.623	0.074
	20–29	125 (30.6)	116.98 ± 23.94		
	30–39	255 (62.3)	123.09 ± 24.89		
	≥40	29 (7.1)	122.41 ± 24.88		
Marital Status	First Marriage	383 (93.6)	120.86 ± 24.55	0.980	0.328
	Remarriage	26 (6.4)	125.77 ± 26.95		
Education Level	Junior high school and below	112 (27.4)	113.80 ± 27.29	7.675	0.001
	High school/vocational school	154 (37.6)	122.49 ± 25.50		
	Bachelor's Degree or Higher	143 (35.0)	125.53 ± 20.15		
Occupation	Unemployed	65 (16.0)	120.89 ± 25.09	2.758	0.042
	Farmer	79 (19.3)	114.27 ± 26.24		
	Self-employed	82 (20.0)	123.49 ± 26.02		
	Enterprise/institution employee	183 (44.7)	123.22 ± 22.85		
Residence	Rural	173 (42.3)	118.52 ± 26.69	1.838	0.067
	Urban	236 (57.7)	123.16 ± 23.04		
Household finances	Income insufficient to cover expenses	64 (15.6)	113.66 ± 25.37	3.830	0.022
	Balanced income and expenditure	305 (74.6)	122.22 ± 23.93		
	Surplus	40 (9.8)	125.23 ± 27.58		
Parenting Experience	None	125 (30.6)	110.15 ± 23.03	6.263	<0.001
	Experience	284 (69.4)	126.03 ± 23.87		

**Table 2:** Univariate analysis of parenting competence in preterm infants according to general demographic and medical conditions.

Variable	Group	*N*(%)	*M* *±* *SD*	*t/F*	*P*
Gender	Male	230 (56.2)	122.96 ± 24.50	1.660	0.098
	Female	179 (43.8)	118.88 ± 24.84		
Gestational Age at Birth				0.122	0.885
	<32weeks	112 (27.4)	120.44 ± 25.58		
	<34weeks	59 (14.4)	120.51 ± 19.58		
	34–37weeks	238 (58.2)	121.69 ± 25.48		
Birth Weight				1.981	0.139
	<1,500g	82 (20.0)	119.61 ± 25.09		
	<2,500g	191 (46.7)	119.40 ± 23.48		
	≥2,500g	136 (33.3)	124.61 ± 25.93		
1-Min Apgar Score*				1.508	0.223
	0–3 points	14 (3.4)	110.86 ± 29.22		
	4–7 points	53 (13.0)	119.36 ± 25.20		
	8–10 points	342 (83.6)	121.88 ± 24.40		
5-Min Apgar Score*				1.600	0.203
	0–3 points	3 (0.7)	96.67 ± 24.95		
	4–7 points	13 (3.2)	118.23 ± 29.84		
	8–10 points	393 (96.1)	121.46 ± 24.49		
Gestational age at discharge				0.906	0.366
	<37 weeks	192 (46.9)	120.00 ± 25.66		
	≥37 weeks	217 (53.1)	122.22 ± 23.82		
Birth weight at discharge				1.485	0.138
	<2,500g	239 (58.4)	119.65 ± 23.96		
	≥2,500g	170 (41.6)	123.32 ± 25.61		
Length of hospital stay				0.298	0.743
	4–19 days	253 (61.9)	121.58 ± 25.08		
	20–39 days	69 (16.9)	121.96 ± 24.41		
	≥40 days	87 (21.2)	119.38 ± 24.00		

* Apgar scores at 1 min and 5 min after birth: 0–3 indicates severe asphyxia; 4–7 indicates mild asphyxia; 8–10 indicates no asphyxia.

### 3.3 Scores of caregiving competence, internal locus of control, and discharge teaching quality

Among the 409 parents of preterm infants, the total caregiving competence score ranged from 49 to 172, with a mean of (121.18 ± 24.70) and a mean item score of (2.82 ± 0.57). The mean scores for each dimension, from highest to lowest, were: Care Attitude (3.33 ± 0.68), Care Behavior (2.91 ± 0.69), and Care Knowledge (2.73 ± 0.69). The internal locus of control scores ranged from 11 to 48 points, with a total score of (34.44 ± 8.34). The discharge teaching quality scores ranged from 43 to 180 points, with a total score of (144.63 ± 31.43) (Table 3).

**Table 3:** 1 scores of caregiving competence, internal locus of control, and discharge teaching quality, as well as the scores of each dimension.

Variable	Score Range	Range of values (points)	*M* ± SD	Average score of entries
Caregiving competence	0–4	49–172	121.18 ± 24.70	2.82 ± 0.57
Care Knowledge	0–4	27–104	70.08 ± 16.40	2.70 ± 0.63
Care giving behavior	0–4	8–48	34.51 ± 7.69	2.88 ± 0.64
Care attitude	0–4	7–20	16.59 ± 3.26	3.32 ± 0.65
Internal locus of control	0–6	11–48	34.44 ± 8.34	4.3 ± 1.04
Discharge teaching quality	0–10	43–180	144.63 ± 31.43	8.03 ± 1.75
Content acquisition	0–10	9–60	47.79 ± 11.59	7.96 ± 1.93
Guidance techniques and effectiveness	0–10	12–120	96.84 ± 21.69	8.07 ± 1.77

### 3.4 Correlations among discharge teaching quality, internal locus of control, and caregiving competence

Pearson correlation analysis showed that discharge teaching quality was positively correlated with caregiving competence (*r* = 0.459, *P* < 0.01) and internal locus of control (*r* = 0.313, *P* < 0.01). Internal locus of control was also positively correlated with caregiving competence (*r* = 0.343, *P* < 0.01) (Table 4).

**Table 4:** Correlation analysis of discharge teaching quality, internal locus of control, and caregiving competence.

Variable	1	2	3
1. Caregiving competence	1		
2. Discharge teaching quality	0.459**	1	
3. Internal locus of control	0.343**	0.313**	1

** *P* < 0.01.

### 3.5 Multivariate linear regression analysis of caregiving competence in parents of preterm infants

With parents' caregiving competence for preterm infants as the dependent variable, the first layer included variables statistically significant in univariate analysis (parental role, educational attainment, occupation, household economic status, parenting experience); the second layer added discharge teaching quality; the third layer incorporated internal locus of control. Results indicate that Model 1 explains 14.2% of the variance in caregiving competence. Adding quality of discharge education increased the model's explanatory power to 28.3%. Further inclusion of internal locus of control elevated the explanatory power to 31.0% (Table 5).

**Table 5:** Multivariate linear regression analysis of factors associated with caregiving competence.

Variable	Model 1		Model 2		Model 3	
	*β*	*P*	*β*	*P*	*β*	*P*
Mother, ref: father^a^	0.153	0.002	0.109	0.018	0.104	0.021
Education level^b^						
High school/vocational school	0.158	0.021	0.137	0.028	0.134	0.028
Bachelor's Degree or Higher	0.234	0.008	0.222	0.006	0.196	0.013
Occupation^c^						
Unemployed	0.049	0.466	0.010	0.867	0.012	0.843
Self-employed	0.005	0.939	0.017	0.792	0.005	0.934
Enterprise/institution employee	0.095	0.290	0.153	0.062	0.168	0.038
Household finances^d^						
Balanced income and expenditure	0.023	0.704	0.016	0.775	0.002	0.973
Surplus	0.022	0.714	0.024	0.666	0.018	0.737
Parenting experience, ref: none^e^	0.284	<0.001	0.210	<0.001	0.185	<0.001
Discharge teaching quality			0.403	<0.001	0.359	<0.001
Internal locus of control					0.177	<0.001
*F*	7.699***		16.427***		16.832***	
*R* ^2^	0.148		0.292		0.318	
△*R*^2^	0.148***		0.144***		0.026***	

*** *P* < 0.001.

a Reference group: fathers.

b Reference group: Junior high school and below.

c Reference group: Farmer.

d Reference group: Income insufficient to cover expenses.

e Reference group: None.

### 3.6 Mediating effect of internal locus of control on the association between discharge teaching quality and caregiving competence

**Figure 1:**
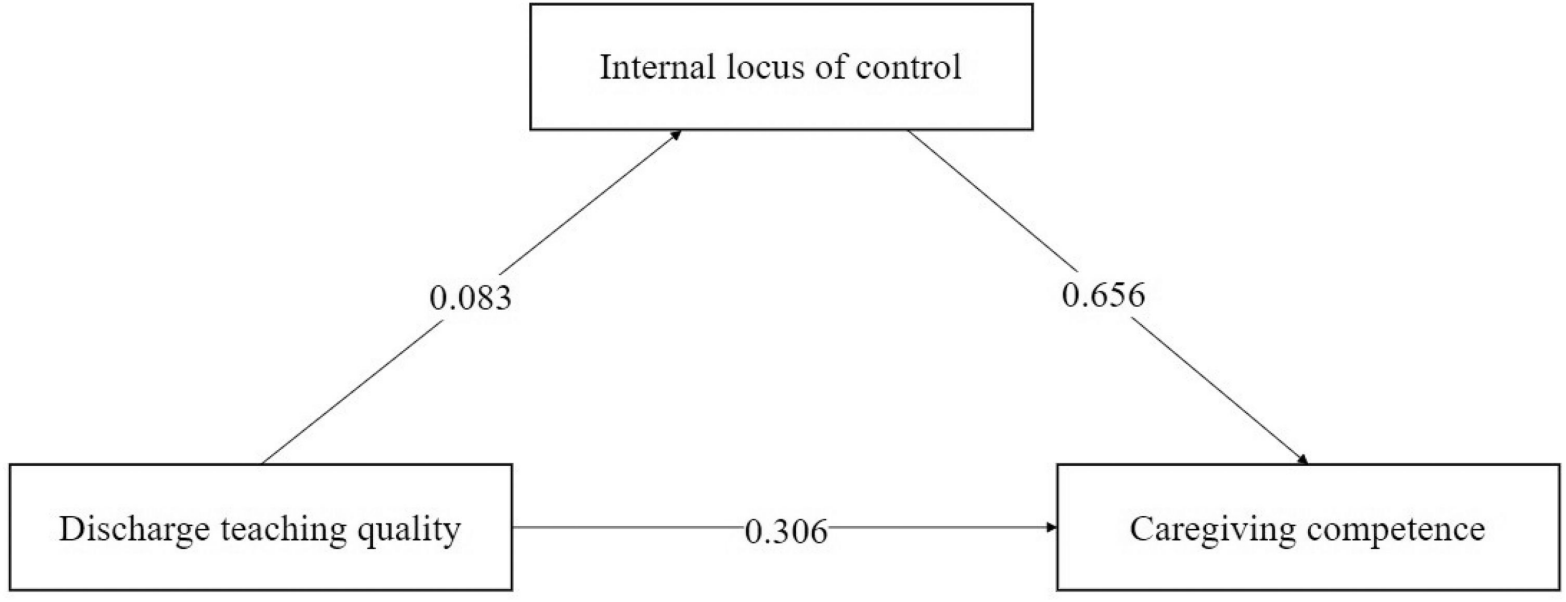
Mediating effect of internal locus of control on the association between discharge teaching quality and caregiving competence.

Using caregiving competence as the dependent variable, discharge teaching quality as the independent variable, and internal locus of control as the mediator, Model 4 in the PROCESS plugin was employed to validate the relationships among variables. Results indicate: Discharge teaching quality had a significant positive total effect on caregiving competence (*β* = 0.360, *SE* = 0.035, *P* < 0.001). It also had a significant direct effect on caregiving competence after adjustment for internal locus of control (*β* = 0.306, *SE* = 0.035, *P* < 0.001). In addition, discharge teaching quality significantly predicted internal locus of control (*β* = 0.083, *SE* = 0.013, *P* < 0.001), and internal locus of control significantly predicted caregiving competence (*β* = 0.656, *SE* = 0.134, *P* < 0.001). The bootstrap results showed a significant indirect effect of 0.054 (*SE* = 0.016, 95% CI: 0.027–0.088), indicating that internal locus of control partially mediated the association between discharge teaching quality and caregiving competence. The indirect effect accounted for 15% of the total effect (Table 6, Figure 1).

**Table 6:** Bootstrap analysis of the mediating effect of internal locus of control.

Project	Estimate	SE	95%CI		Effect size (%)
			Lower	Upper	
Total effect	0.360	0.035	0.293	0.429	100%
Direct effect	0.306	0.035	0.237	0.376	85%
Indirect Effect	0.054	0.016	0.027	0.088	15%

## 4 Discussion

### 4.1 Caregiving competence among parents of preterm infants: current status and associated factors

This study found that the caregiving competence of parents of preterm infants at NICU discharge was at a moderate level, indicating considerable room for improvement. This finding is generally consistent with that of Zhao et al. (5). Among the three dimensions, scores ranked from highest to lowest as follows: caregiving attitude, caregiving behavior, and caregiving knowledge. This suggests that although parents generally hold positive caregiving attitudes, their caregiving knowledge remains insufficient. In particular, most parents have only basic daily care skills and lack professional knowledge and practical skills related to preterm infant care. Several factors may explain this finding. First, limited parental access to the NICU may reduce opportunities for bedside participation, thereby limiting parents' acquisition of caregiving knowledge and practical skills. Second, preterm infants are physiologically immature and medically vulnerable. They are prone to apnea, feeding intolerance, infection, and other complications related to prematurity (2, 3), which requires parents to master more specialized caregiving knowledge and skills. In addition, parents of NICU infants commonly experience anxiety, depression, and a heavy caregiving burden (22, 23). Excessive caregiving burden may hinder caregivers' ability to improve their caregiving skills (24). This study also found that parental role, educational attainment, and parenting experience were significantly associated with parental caregiving competence, which is consistent with the findings of Huang et al. (25). Mothers demonstrated higher caregiving competence than fathers, possibly because mothers' greater involvement in daily infant care in the local sociocultural context. Higher educational attainment was also associated with higher caregiving competence, suggesting that education may enhance parents' ability to understand and apply caregiving knowledge. In addition, parents with prior parenting experience had higher caregiving competence scores, which may be attributed to their greater familiarity with infant care and their ability to manage basic caregiving issues more effectively.

### 4.2 Direct association between discharge teaching quality and caregiving competence among parents of preterm infants

This study found that discharge teaching quality was positively associated with caregiving competence, consistent with previous studies (26, 27). This association remained significant after adjustment for relevant covariates, suggesting that high-quality discharge teaching may play an important role in enhancing parents' competence in caring for preterm infants at home. Given the unique characteristics and vulnerability of preterm infants, parents are required not only to provide basic daily care but also to acquire specialized knowledge and skills related to preterm infant care, such as emergency management of aspiration and choking and nutritional management strategies. Timely, high-quality discharge teaching addresses the diverse care needs of parents by providing essential caregiving knowledge and relevant coping strategies, thereby enhancing their caregiving competence (28). In clinical practice, nurses can provide personalized health education tailored to the child's parents' age, educational background, occupation, geographic location, family financial situation, and parenting experience. Through hands-on demonstrations and simulation exercises, nurses assist parents in mastering home care skills related to the scientific feeding of premature infants, proper medication administration, vital sign monitoring, and early observation of the child's condition. In addition, effective discharge teaching may reduce uncertainty during the transition from NICU to home care and help parents apply caregiving skills more confidently in daily practice.

### 4.3 Association between internal locus of control and caregiving competence

The findings of this study confirmed the hypothesis that internal locus of control was positively correlated with caregiving competence. Parents with higher internal locus of control had better caregiving competence, consistent with previous studies (10–12). The locus of control, derived from social learning theory, refers to an individual's causal beliefs and perceptions regarding whether health outcomes are controlled by their own behavior or by external factors (14). Parents with a stronger internal locus of control are more likely to believe that their own actions can influence their infant's condition and development. Therefore, they may be more motivated to seek information, participate in parenting training, and practice caregiving skills. In addition, individuals with stronger internal locus of control often show better emotional regulation and are more likely to use positive coping strategies when facing stressful situations (29, 30). By contrast, parents with lower internal locus of control may attribute caregiving difficulties to luck, medical conditions, or other external factors, which may reduce their motivation to learn actively and hinder their adaptation to the caregiving role. These findings suggest that internal locus of control is an important psychological factor associated with caregiving competence among parents of preterm infants.

### 4.4 The mediating role of internal locus of control between discharge teaching quality and caregiving competence

The findings indicated that internal locus of control partially mediated the relationship between discharge teaching quality and caregiving competence. In other words, discharge teaching quality was associated with caregiving competence both directly and indirectly through internal locus of control. These findings can be understood through transition theory, which suggests that the transition process and its outcomes are influenced by multiple factors, including individual characteristics, environmental factors, and nursing interventions. In this study, discharge teaching quality can be viewed as an important nursing intervention that facilitates this transition, while internal locus of control reflects the extent to which parents feel able to participate actively and manage the caregiving role. Specifically, clear, practical, personalized, and easy-to-understand discharge teaching helps parents acquire caregiving knowledge and skills more effectively. In addition, high-quality discharge teaching provides psychological support and coping strategies to help parents alleviate stress associated with the transition period (29, 30). In this process, high-quality discharge teaching serves not only to convey information but also as a means to help parents build confidence, enhance their sense of control, adapt to their role as caregivers at home, and strengthen their belief that their own efforts can improve their infant's health and developmental outcomes. From the perspective of transition theory, this affirmation of personal agency reflects parents’ active participation, perceived sense of control, and role mastery during the transition process—key psychological mechanisms that facilitate a healthy transition. Therefore, internal locus of control may serve as an important psychological pathway linking discharge teaching quality to caregiving competence.

### 4.5 Practical significance

These findings have important implications for clinical nursing practice. Discharge teaching for parents of preterm infants should not be limited to routine information delivery but should also support their psychological adaptation to the home caregiving role after NICU discharge. Nursing staff should encourage parents to express their needs, concerns, and difficulties in a timely manner, so as to help them build confidence and alleviate emotional stress. In particular, early assessment of parents' locus of control, caregiving confidence, emotional status, and readiness for home care is essential for providing targeted support. For parents with lower internal locus of control, nursing staff should provide additional support aimed at strengthening their perceived control and confidence in caregiving. This may include supportive communication, individualized education, demonstration-based teaching, the teach-back method, and positive feedback. These approaches may help parents recognize that their own caregiving behaviors can positively influence their infant's health and development, thereby promoting more active participation in care. For parents with higher internal locus of control, nursing staff should continue to provide affirmation, reinforcement, and individualized guidance according to their specific needs, so as to further support their active involvement in infant care. In addition, health education should be delivered through diverse formats and channels tailored to the characteristics and needs of parents of preterm infants. Educational approaches should emphasize a step-by-step progression, avoiding overwhelming parents with excessive information in a single session. This enables better absorption and application of newly acquired knowledge and skills, thereby promoting the development of caregiving competence.

### 4.6 Limitations

This study has several limitations. First, because this was a cross-sectional study, causal relationships among discharge teaching quality, internal locus of control, and caregiving competence could not be established. Future longitudinal or intervention studies are needed to further clarify the causal pathways among these variables. Second, participants were recruited from the NICU of a single hospital, resulting in a geographically limited sample. Multi-center studies with larger sample sizes are needed to further verify the generalizability and robustness of the findings. Third, this study only examined the mediating role of internal locus of control in the relationship between discharge teaching quality and caregiving competence. Other psychological, family, and social factors may also influence this relationship and future research could explore additional potential mediating or moderating factors. Finally, the sample was restricted to two-parent families in which both parents were involved in caregiving; therefore, the results may not be generalizable to single-parent families, divorced or separated families, or families in which a grandparent or another guardian is the primary caregiver.

## 5 Conclusion

This study developed a mediation model based on transition theory, providing deeper insight into the mechanism underlying the relationship between discharge teaching quality and caregiving competence among parents of preterm infants. The findings indicate that internal locus of control partially mediates the relationship between discharge teaching quality and caregiving competence. Therefore, when delivering discharge teaching, healthcare providers should focus on enhancing parents' internal locus of control through participatory guidance and contextualized teaching. Encouraging parents to actively ask questions and restate key points, while healthcare providers promptly offer affirmation and correction, helps parents develop controllable and predictable caregiving beliefs and action strategies. This may help parents develop the capability and confidence to independently and consistently provide care upon discharge, thereby potentially improving their caregiving competence.

## Data Availability

The raw data supporting the conclusions of this article will be made available by the authors, without undue reservation.
